# Comparison of Virtual Non-Contrast and True Non-Contrast CT Images Obtained by Dual-Layer Spectral CT in COPD Patients

**DOI:** 10.3390/bioengineering11040301

**Published:** 2024-03-22

**Authors:** Manuel Steinhardt, Alexander W. Marka, Sebastian Ziegelmayer, Marcus Makowski, Rickmer Braren, Markus Graf, Joshua Gawlitza

**Affiliations:** Department of Diagnostic and Interventional Radiology, School of Medicine & Klinikum rechts der Isar, Technical University of Munich, 81675 Munich, Germany; alexander.marka@tum.de (A.W.M.); s.ziegelmayer@tum.de (S.Z.); marcus.makowski@tum.de (M.M.); rbraren@tum.de (R.B.); markus.m.graf@tum.de (M.G.)

**Keywords:** COPD, computed tomography imaging, virtual non-contrast images, true non-contrast images, dual-layer spectral CT, emphysema quantification, lung imaging, radiation exposure, image comparison analysis

## Abstract

Chronic obstructive pulmonary disease (COPD) is one of the leading causes of death. Recent studies have underlined the importance of non-contrast-enhanced chest CT scans not only for emphysema progression quantification, but for correlation with clinical outcomes as well. As about 40 percent of the 300 million CT scans per year are contrast-enhanced, no proper emphysema quantification is available in a one-stop-shop approach for patients with known or newly diagnosed COPD. Since the introduction of spectral imaging (e.g., dual-energy CT scanners), it has been possible to create virtual non-contrast-enhanced images (VNC) from contrast-enhanced images, making it theoretically possible to offer proper COPD imaging despite contrast enhancing. This study is aimed towards investigating whether these VNC images are comparable to true non-contrast-enhanced images (TNC), thereby reducing the radiation exposure of patients and usage of resources in hospitals. In total, 100 COPD patients with two scans, one with (VNC) and one without contrast media (TNC), within 8 weeks or less obtained by a spectral CT using dual-layer technology, were included in this retrospective study. TNC and VNC were compared according to their voxel-density histograms. While the comparison showed significant differences in the low attenuated volumes (LAVs) of TNC and VNC regarding the emphysema threshold of −950 Houndsfield Units (HU), the 15th and 10th percentiles of the LAVs used as a proxy for pre-emphysema were comparable. Upon further investigation, the threshold-based LAVs (−950 HU) of TNC and VNC were comparable in patients with a water equivalent diameter (DW) below 270 mm. The study concludes that VNC imaging may be a viable option for assessing emphysema progression in COPD patients, particularly those with a normal body mass index (BMI). Further, pre-emphysema was generally comparable between TNC and VNC. This approach could potentially reduce radiation exposure and hospital resources by making additional TNC scans obsolete.

## 1. Introduction

Chronic obstructive pulmonary disease (COPD) is a common and largely avoidable disease that is characterized by persistent respiratory symptoms and irreversible airway obstruction. It usually develops slowly over years in response to smoking, has an increased incidence with an older age and after years of exposure to inhaled noxae and particles, it is characterized by chronic bronchitis and emphysema of the lung [1,2]. 

In recent years, quantitative computed tomography (qCT) has gained more relevance in the evaluation of disease progression in COPD patients by detecting and quantifying volumes of low attenuation (LAVs), representing emphysema in CT images [3]. There are different approaches to quantifying LAV areas. The most used parameter today is a threshold of −950 HU, which has been found to ensure the strongest correlation of qCT and emphysema on a microscopic and macroscopic level [4,5]. Another approach, which is considered as more robust to changes in lung volume in longitudinal studies, is to evaluate the 10th or the 15th percentile of a frequency histogram of lung attenuation [4,6]. 

It has been shown that specific pathological findings in non-contrast-enhanced chest CT images in patients suffering from COPD, such as the extent of emphysema and airway abnormality, are good predictors of COPD exacerbation, as well as mortality [7,8,9]. In response to these discoveries, the American thoracic society and the European respiratory society proposed routine chest CTs for patients clinically diagnosed with COPD [10]. To accurately assess the extent of emphysema and, consequently, the state of COPD, non-contrast-enhanced CT images are needed. Intravenous contrast application has been shown to result in a higher density of lung parenchyma, thus decreasing the volume classified as emphysema in qCT [11]. However, in daily clinical practice, contrast-enhanced chest CT is performed in most patients to diagnose or exclude several vascular, pulmonary, or malignant diseases [12,13,14]. Regarding COPD patients and the named recommendations on non-contrast-enhanced scans, this would result in potential double examinations with and without contrast media. 

Since the introduction of spectral CT scanners equipped with the appropriate software packages into the clinical field, it has been possible to routinely calculate virtual non-contrast-enhanced CT images from contrast-enhanced CT images [15,16]. It was previously shown that generated VNC images from clinically acquired data through a dual-layer CT system were comparable in most tissues, but not bone [16]. 

This advance in CT scanner and software technology could also offer the possibility to assess the disease progression of COPD patients retrospectively from previously performed contrast-enhanced CT images with the help of dual-layer spectral CT-scanners, thereby reducing the radiation exposure of the patients on the one hand and the deployed resources by the hospital on the other. To evaluate this promising approach for further dual-energy techniques, this study sheds light on the comparison of virtual non-contrast-enhanced chest CT images (VNC) with true non-contrast chest CT images (TNC) obtained by a dual-layer spectral CT in patients with COPD. 

## 2. Materials and Methods

Institutional review board approval was obtained for this retrospective study, including all its protocols (protocol number: 180/17S, date of approval: 9 May 2017). Informed consent was waived by the institutional review board, as no additional data besides clinical obtained images were used. All examinations were performed exclusively for diagnostic use to full extent and were performed only with clinical standard protocols. All patient data were completely anonymized at the beginning of the study.

In this study 100 patients, from 18 to 85 years of age (mean = 56.41 years), 44 females (19–85 years, mean = 57.68 years) and 56 males (18–84 years, mean = 55.41 years), were retrospectively enrolled. The inclusion criteria besides diagnosed COPD were: (1) CT scans on the same scanner within eight weeks or less, and (2) one of which must be non-contrast and one contrast-enhanced. Scans with severe effusions, consolidations, or infiltrates, which distorted the emphysema characteristics, were excluded. No preselection regarding age, sex, weight, or other characteristics was performed. The main indications for examinations were the exclusion of pulmonary embolism, evaluation of COPD progression due to clinical deterioration of the respective patients, and screening for lung cancer.

The mean size specific dosage estimate (SSDE) for all native images was 3.95 ± 2.16 mGy, while the mean SSDE for all contrast images was 5.67 ± 2.06 mGy.

In each patient, a true non-contrast (TNC) image was compared to a virtual non-contrast (VNC) image. The images were obtained exclusively with a 64-slice single-source dual-layer spectral CT system (IQon; Philips Healthcare, Cleveland, OH, USA). The VNC images were created with the help of the vendor-specific spectral workstation (IntelliSpace Portal (v. 8.0.2)). The time difference between the images was, on average, 16.83 ± 11.83 days (range: 0–54 days). All datasets, TNC images, and VNC images were reconstructed in axial view with a slice thickness of 0.9 mm. Thus, they were identical regarding the image positioning and orientation. In the case of the VNC images created from contrast-enhanced images, the contrast medium (Ultravist 370 MCT, Bayer Vital GmbH, Leverkusen, Germany) volume was 73.25 ± 14.70 mL, resulting in an iodine amount of 27.34 ± 5.87 g. The contrast CTs were either in the pulmonary arterial phase (21 patients) or in the portal venous phase (79 patients).

**Figure 1:**
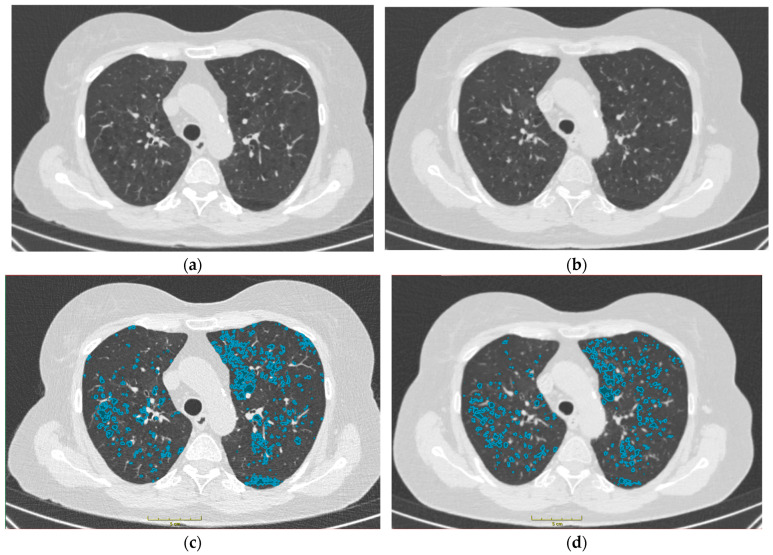
Comparison of TNC and VNC images and comparison of respective emphysema segmentations from a patient included in this study. (**a**) TNC. (**b**) VNC. (**c**) TNC + Segmentation. (**d**) VNC + Segmentation. Emphysema = Lung parenchyma < −950 HU. HU = Houndsfield units. Segmentation was done with ImFusion Labels 0.19.3 (ImFusion GmbH, Munich, Germany).

Both the TNC and VNC images were analyzed with the COPD tool from Philips IntelliSpace (IntelliSpace Portal (v. 8.0.2), Philips Healthcare, Cleveland, OH, USA) (Figure 1). In all datasets, the noise suppression function was used. From the resulting voxel density histogram, a low attenuated volume (LAV) below the threshold of −950 HU, as well as the 10th and 15th percentiles, were used to compare TNC and VNC images, as they have previously been reported to be indicators for emphysema and pre-emphysema, respectively [4,6]. 

The following possible influencing factors on LAV were investigated: sex, age, contrast phase, total iodine amount in contrast medium, iodine to weight ratio, time difference between TNC and VNC images, BMI, water equivalent diameter (DW), and SSDE.

The DW was obtained from the TNC images using an open-source code based on the official formula of the AAPM Task Group 220, which returns a specific DW value for one slice of the respective CT image [17,18]. This code was modified by the authors to automatically perform the same process for all slices of the image. The mean of all slices was consequently used as the DW in this study. The SSDE was calculated multiplying CTDIvol with a conversion factor, which is dependent on the DW value [18]. 

All statistical analyses were performed using a dedicated software package (Prism 9.3.1, GraphPad Software, San Diego, CA, USA). The data were tested for Gaussian distribution via a D’Agostino–Pearson omnibus test. The Wilcoxon-test and Mann–Whitney U test were used when groups showed a non-Gaussian distribution. All variables were continuous and are expressed as median and interquartile range. A *p* value < 0.05 was considered to be statistically significant. Furthermore, n represents the number of observed pairs, while r was calculated to determine the effectiveness of the pairing. When the total amount of LAV in TNC and VNC was compared, a Bland–Altman Plot was used to better visualize the results.

## 3. Results

### 3.1. Comparison of Emphysema in TNC and VNC Images

**Figure 2:**
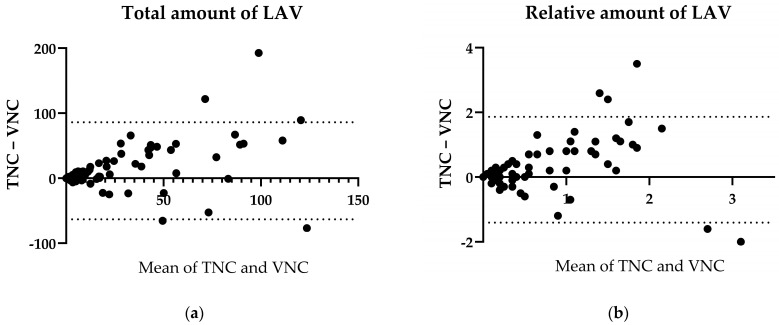
Emphysema. (**a**) Bland–Altman plot of total amount of LAV in TNC and VNC. (**b**) Bland–Altman plot of relative amount of LAV in TNC and VNC.

LAV is defined by the volume of the lungs which is classified as emphysema, namely, the sum of all voxels with −950 HU and less. When the total amounts of LAV in the TNC and VNC images throughout the whole cohort were compared, there was a significantly lower total amount in the VNC group, with a median of differences of −2.75 cm^3^ (*p* < 0.0001, r = 0.79, n = 98, Figure 2a). Similarly, the relative amount of LAV regarding the total lung volume was significantly lower in the VNC than in the TNC images, with a median of differences of −0.1% (*p* = 0.0003, r = 0.75, n = 77, Figure 2b). 

To further investigate the discrepancies between the total amounts of LAV in the TNC and VNC images, several possible underlying confounders were analyzed.

**Figure 3:**
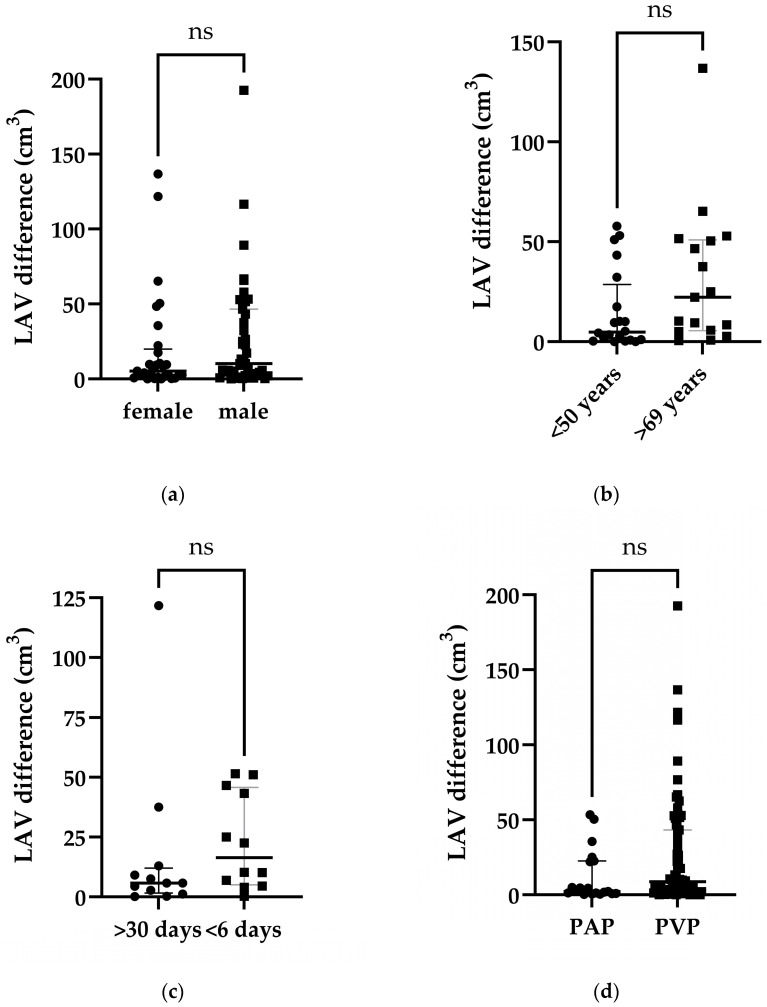

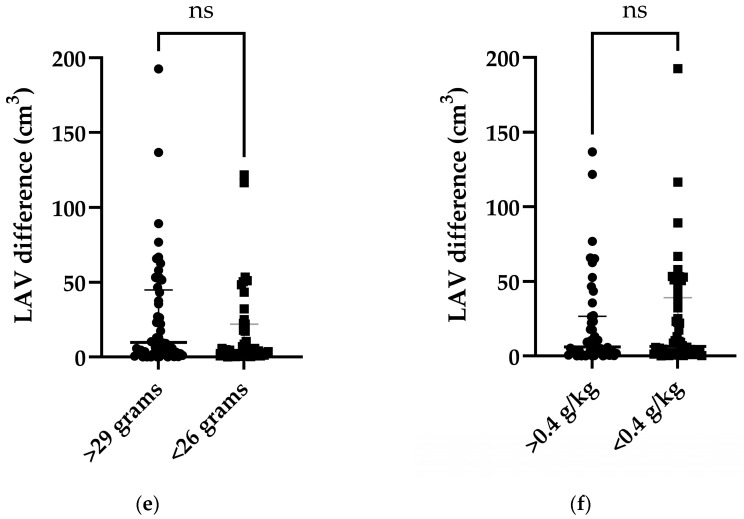
Scatter plots of LAV differences between TNC and VNC images for the following investigated possible confounders: (**a**) Sex. (**b**) Age. (**c**) Time difference between images. (**d**) Phase of contrast. (**e**) Iodine amount. (**f**) Iodine/weight ratio. Data are shown with median (big stroke) and interquartile range (small strokes) in the graph. ns = no significant difference between medians of both groups (*p* value > 0.05). PAP = Pulmonary arterial phase. PVP = Portal venous phase.

The median LAV differences between the TNC and VNC images in males (n = 43) and females (n = 29) were 10.30 cm^3^ and 5.20 cm^3^, respectively. Both groups were comparable (U = 491 *p* = 0.1295, Figure 3a). Comparing older (>69 years, n = 17) and younger patients (<50 years, n = 20) revealed median LAV differences of 20.30 cm^3^ and 4.85 cm^3^, and as between the sexes, no significant age differences were shown (U = 111.50, *p* = 0.0756, Figure 3b). Furthermore, the time difference between the acquisition of the particular TNC and VNC image, (U = 44.05, *p* = 0.1169, Figure 3c), the phase of contrast, (U = 563, *p* = 0.1065, Figure 3d), the total iodine amount, (U = 979, *p* = 0.1235, Figure 3e), and the iodine/weight ratio (U = 1172, *p* = 0.6285, Figure 3f) did not significantly influence the LAV differences between the TNC and VNC images. 

**Figure 4:**
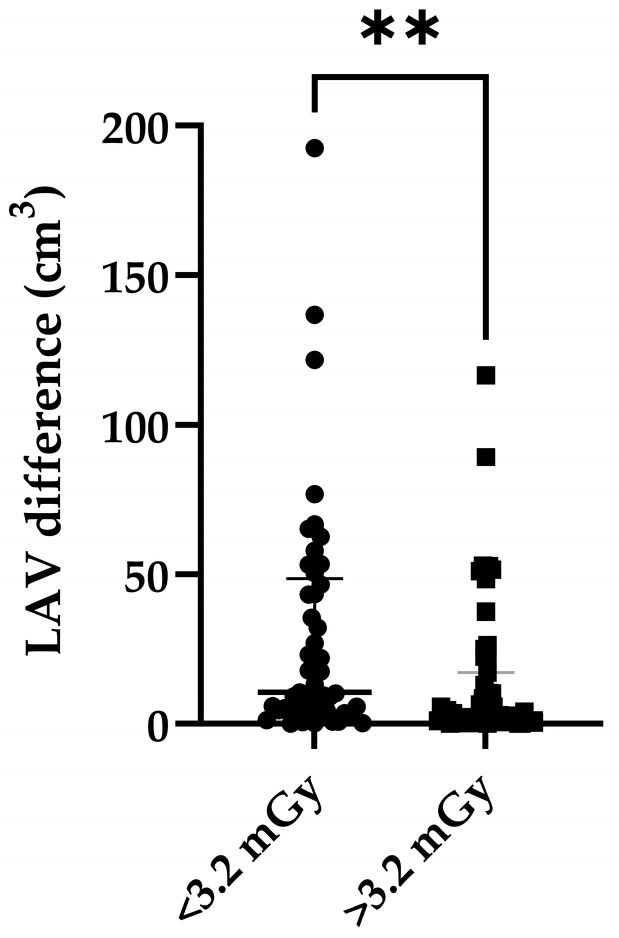
Scatter plot of LAV differences between TNC and VNC images regarding size-specific dosage estimate of TNC images. Data are shown with median (big stroke) and interquartile range (small strokes) in the graph. ** = significant difference between medians of both groups (*p* value ≤ 0.01).

There were, however, significantly higher LAV differences between the TNC and VNC images in patients with a size-specific dose estimate below 3.2 mGy (n = 49, median = 10.5 cm^3^) when compared to patients with a size-specific dose estimate of more than 3.2 mGy (n = 51, median = 3.2 cm^3^, U = 809, *p* = 0.0022) (Figure 4). The size-specific dose estimate is a radiation dose that takes a patient’s size into account.

**Figure 5:**
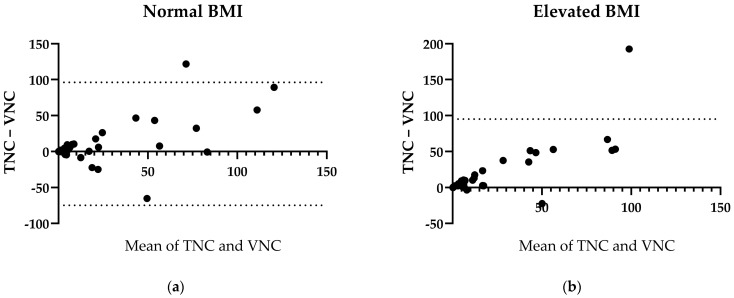
LAV Differences between TNC and VNC regarding body mass index. (**a**) Bland–Altmann plot of normal BMI. (**b**) Bland–Altmann plot of elevated BMI. BMI = Body mass index (kg/m^2^). Normal = 18.5–24.9 kg/m^2^. Elevated = >25 kg/m^2^.

BMI and DW were used as proxies for the body composition of the patients. Patients with a normal BMI (18.5–25 kg/m^2^) showed borderline significant differences in the total LAV in the TNC and VNC images, with a median of differences of −4 cm^3^ (*p* = 0.0425, r = 0.83, n = 35, Figure 5a). Patients with an elevated BMI (>25 kg/m^2^), on the other hand, displayed more distinct differences between the total amount of LAV in the TNC and VNC images, with a median of differences of −5.5 cm^3^ (*p* < 0.0001, r = 0.83, n = 34, Figure 5b). 

**Figure 6:**
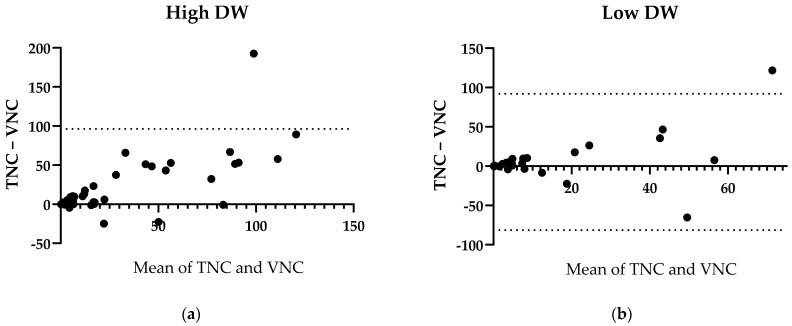
LAV Differences between TNC and VNC regarding water equivalent diameter. (**a**) Bland–Altmann plot of high DW. (**b**) Bland–Altman plot of low DW. DW = Water equivalent diameter. High = >270 mm. Low = <270 mm.

The DW was calculated to obtain a more objective parameter of body composition, since BMI is mostly based on estimations of weight and height by medical technical assistants in daily clinical practice. The total amount of LAV in patients with a DW > 270 mm was significantly higher in the TNC images compared to the respective VNC images, with a median of differences of =−5.9 cm^3^ (*p* < 0.0001, r = 0.74, n = 41, Figure 6a). Contrariwise, the LAVs in the TNC and VNC images in patients with a DW < 270 mm were comparable, with a median of differences of =−2.8 cm^3^ (*p* = 0.1722, r = 0.83, n = 30, Figure 6b).

To evaluate the applicability of the VNC images created by dual-layer spectral CT systems to the quantification of emphysema in COPD patients, the total amount of LAV of these images was compared to the total amount LAV of the TNC image from the same patient. Due to significant differences in the total amounts of LAV in the TNC and VNC images, several possible underlying confounders, namely age, sex, BMI, SSDE, total amount of iodine, iodine/weight ratio, time difference between VNC and TNC image, and contrast phase, were investigated. BMI has been shown to influence the comparability of TNC and VNC images regarding the total amount of LAV. Upon further analysis, it was demonstrated that the TNC and VNC images of patients with a normal BMI showed only borderline significant differences, while in patients with an elevated BMI, more distinct differences in the LAVs of TNC and VNC images had been shown. To confirm the role of body composition in the comparability of TNC and VNC images regarding emphysema quantification by a more objective approach, the water equivalent diameter was calculated.

### 3.2. Comparison of Pre-Emphysema in TNC and VNC Images

The 10th and 15th percentiles of a frequency histogram of lung attenuation were employed as proxies for pre-emphysema [4,6]. 

**Figure 7:**
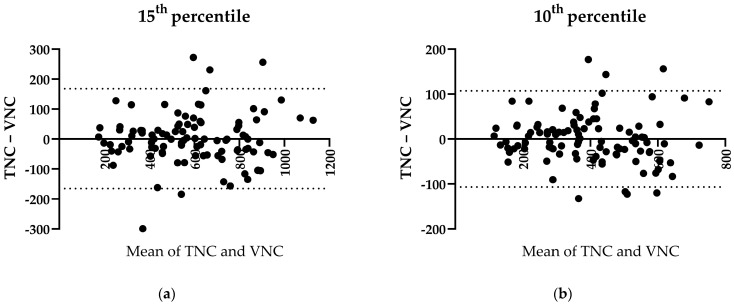
Pre-emphysema. (**a**) Bland–Altman plot of 15th percentile of LAV in TNC and VNC. (**b**) Bland–Altman plot of 10th percentile of LAV in TNC and VNC.

The amounts of LAV were comparable between the TNC and VNC images on both occasions, for the 10th percentile (median of differences = 5.85 cm^3^, *p* = 0.7160, r = 0.95, n = 100, Figure 7b) and the 15th percentile (median of differences = 0.75 cm^3^, *p* = 0.9121, r = 0.93, n = 100, Figure 7a). 

## 4. Discussion

In the present study, VNC images generated from contrast-enhanced arterial- and portal-venous-phase scans obtained by a dual-layer spectral computed tomography system were compared to TNC images in COPD patients regarding their accuracy in determining the extent of lung emphysema and pre-emphysema. As our results show, TNC and VNC emphysema quantification is comparable, as no significant differences were found in the TNC and VNC images from patients with a comparable body composition (DW < 270 mm). 

VNC imaging has been introduced into clinical routine as an application of spectral data from DE-CT systems, and it has been shown that it can replace TNC images in multiple clinical settings [19]. However, one major limitation of current DE-CT systems is the prerequisite that examinations must be designed prospectively. The retrospective acquisition of spectral data and, therefore, the generation of VNC images are not possible with the dual-energy technique. With dual-layer CT, however, this major downside is overcome, as spectral data are acquired by default. Hence, data that have been acquired previously can be used to reconstruct VNC images.

However, when assessing disease progression for emphysema characterized by an LAV lower than −950 HU in the whole cohort, the LAVs in the TNC and VNC images were not comparable. The reason for this may have been attenuation of the X-rays in patients with a higher BMI or DW, respectively, due to excessive soft tissue [20]. This argument is further supported by the finding that the TNC and VNC images in patients with a higher SSDE and, therefore, stronger X-ray penetration showed significantly lower LAV differences than patients with low SSDE exposure.

In regard to the comparability of the dual-layer spectral CT system used in this study, there are no studies yet comparing VNC images in the lung with the various dual-energy modalities. However, other image quality aspects of the different dual-energy systems were investigated. It was found that iodine and monochromatic accuracy varies among systems [21] and that dual-layer CT, in contrast to other systems, increases diagnostic information, at least in abdomen CT scans, through low noise and high image contrast [22].

In addition to our approach using qCT to test the comparability of TNC and VNC images regarding the total amount of LAV, the employment of machine learning models in emphysema quantification led to a greater prediction accuracy of subsequent health service use by the patients [23] on the one hand, and to a better assessment of individuals at risk through heterogenous structural changes in their lungs using qCT data on the other [24]. Together with our findings, these approaches could further improve the prediction of emphysema progression in COPD patients, while simultaneously reducing the financial burden of the healthcare system through a more effective distribution of resources.

The patient collective in our study was representative with respect to age and sex, comprising 56% male patients and 44% female patients with an average age of 56.41 years. Male preponderance and older age in patients regarding disease prevalence are well-established [25]. Furthermore, our collective contained only 48% patients that were either overweight or obese (BMI > 25 kg/m^2^), while some studies have shown that the majority of adults in Germany have an elevated BMI [26]. This slight difference could be explained by findings in other studies regarding a lower prevalence of COPD in patients with a BMI of 24.9 or higher [25]. 

The retrospective design of our study is one of its limitations. Hence, confounders must be considered, as well as the possibility of the sample not being perfectly comparable. Furthermore, the findings in the present study are only valid for spectral data obtained and VNC images reconstructed by the 64-slice single-source dual-layer spectral CT system with the respective software package. Different spectral imaging approaches, especially the current developments in photon counting, might differ in their correlating findings. Additionally, in the case of patients with a higher DW or BMI, either a conversion factor must be investigated or higher radiation dosages should be applied to these patients, since SSDE seems to influence the proper creation of VNC images. Lastly, the sample of this study comprised a high number of patients with only small total volumes of emphysema, which probably led to its non-Gaussian distribution.

A big advantage of the findings in this study is that patients with a normal body composition who undergo a contrast-enhanced CT-scan with a dual-layer system, for various reasons, can be assessed simultaneously for their emphysema progression. Since non-contrast-enhanced CT scans are crucial in emphysema assessment [11], this approach can drastically reduce the patients’ radiation exposure, as well as the hospitals’ resources.

In practical applications, our results suggest the possibility of simultaneously assessing lung emphysema while patients are examined for other diseases, especially malign entities such as lung cancer, which are associated with smoking and, therefore, COPD [27,28]. 

Future studies should shed light on the comparability between different spectral imaging systems, as well as different manufacturers, in the assessment of lung emphysema. 

## Data Availability

The data presented in this study are available on request from the corresponding author due to privacy reasons.
